# Supplementary material for: A Critical View of the Application of the APEX Software (Aqueous Photochemistry of Environmentally-Occurring Xenobiotics) to Predict Photoreaction Kinetics in Surface Freshwaters
Source: Molecules. 2019 Dec 18;25(1):9. doi: 10.3390/molecules25010009 (PMC7017383; doi:10.3390/molecules25010009)
Supplement: Supplementary file 1 [file molecules-25-00009-s001.zip › 3.2.4_gcc-4.4.0.zip!/3.2.4_gcc-4.4.0/3.2.4_gcc-4.4.0/doc/octave/html/interpreter/Call-by-Value.html]

Call by Value - Untitled


Next: Recursion,
Up: Calling Functions


---

#### 8.2.1 Call by Value

In Octave, unlike Fortran, function arguments are passed by value, which
means that each argument in a function call is evaluated and assigned to
a temporary location in memory before being passed to the function.
There is currently no way to specify that a function parameter should be
passed by reference instead of by value. This means that it is
impossible to directly alter the value of a function parameter in the
calling function. It can only change the local copy within the function
body. For example, the function

```
     function f (x, n)
       while (n-- > 0)
         disp (x);
       endwhile
     endfunction
```

displays the value of the first argument n times. In this
function, the variable n is used as a temporary variable without
having to worry that its value might also change in the calling
function. Call by value is also useful because it is always possible to
pass constants for any function parameter without first having to
determine that the function will not attempt to modify the parameter.

The caller may use a variable as the expression for the argument, but
the called function does not know this: it only knows what value the
argument had. For example, given a function called as

```
     foo = "bar";
     fcn (foo)
```

you should not think of the argument as being “the variable
`foo`.” Instead, think of the argument as the string value,
`"bar"`.

Even though Octave uses pass-by-value semantics for function arguments,
values are not copied unnecessarily. For example,

```
     x = rand (1000);
     f (x);
```

does not actually force two 1000 by 1000 element matrices to exist
*unless* the function `f` modifies the value of its
argument. Then Octave must create a copy to avoid changing the
value outside the scope of the function `f`, or attempting (and
probably failing!) to modify the value of a constant or the value of a
temporary result.
